# A New Model Including AMH Cut-off Levels to Predict Post-treatment Ovarian Function in Early Breast Cancer: A Prospective Cohort Study

**DOI:** 10.34172/aim.2024.15

**Published:** 2024-02-01

**Authors:** Ramesh Omranipour, Fatemeh Ahmadi-Harchegani, Azin Saberi, Ashraf Moini, Mostafa Shiri, Amirmohsen Jalaeefar, Arvin Arian, Akram Seifollahi, Mahshad Madani, Bita Eslami, Sadaf Alipour

**Affiliations:** ^1^Breast Diseases Research Center (BDRC), Cancer Institute, Faculty of Medicine, Tehran University of Medical Sciences, Tehran, Iran; ^2^Department of Oncologic Surgery, Cancer Institute, Faculty of Medicine, Tehran University of Medical Sciences, Tehran, Iran; ^3^Cancer Control Research Center, Cancer Control Foundation, Iran University of Medical Sciences, Tehran, Iran; ^4^Department of Biostatistics, Faculty of Health, Tehran University of Medical Sciences, Tehran, Iran; ^5^Department of Surgery, Arash Women’s Hospital, Faculty of Medicine, Tehran University of Medical Sciences, Tehran, Iran; ^6^Department of Infertility, Arash Women’s Hospital, Faculty of Medicine, Tehran University of Medical Sciences, Tehran, Iran; ^7^Department of Endocrinology and Female Infertility at Reproductive Biomedicine Research Center, Royan Institute for Reproductive Biomedicine, ACECR, Tehran, Iran; ^8^Faculty of Mathematical Sciences, Shahid Beheshti University, Tehran, Iran; ^9^Department of Radiology, Cancer Institute, Faculty of Medicine, Tehran University of Medical Sciences, Tehran, Tehran, Iran; ^10^Department of Pathology, Arash Women’s Hospital, Faculty of Medicine, Tehran University of Medical Sciences, Tehran, Iran; ^11^Faculty of Medicine, Tehran University of Medical Sciences, Tehran, Iran

**Keywords:** Amenorrhea, Anti-mullerian hormone, Breast cancer, Chemotherapy, Fertility preservation

## Abstract

**Background:**

Breast cancer (BC) treatment decreases fertility capacity, but unnecessary fertility preservation procedures in women who would not be infertile after treatment would be a waste of time and resources and could cause the unwarranted exposure of cancer cells to exogenous sex hormones. It has been largely shown that post-treatment ovarian reserve is directly associated with pre-treatment anti-mullerian hormone levels (AMH0). A threshold for AMH0, or a model including AMH0 and patient characteristics that could distinguish the patients who will be infertile after treatments, still needs to be defined. Accordingly, this study was performed to specifically target this high-priority concern.

**Methods:**

Women≤45 years old with newly diagnosed non-metastatic BC were entered in this multicenter prospective cohort study. AMH0 and two-year post-treatment AMH (AMH2) were measured, and hormonal patient features were recorded as well. *Receiver operating characteristic* (ROC) curve analysis, decision tree (DT), and random forest analyses were performed to find a cut-off point for AMH0 and define a model involving related features for the prediction of AMH2.

**Results:**

The data from 84 patients were analyzed. ROC curve analysis revealed that AMH0>3 ng/mL (Area under the curve=0.69, 95% CI: 0.54‒0.84) was the best indicator for predicting AMH2≥0.7 (sensitivity=79%, specificity=60%). The best model detected by DT and random forest for predicting an AMH2>0.7 with a probability of 93% consisted of a combination of AMH0>3.3, menarche age<14, and age<31.

**Conclusion:**

This combination model can be used to withhold fertility preservation procedures in BC patients. Performing larger studies is suggested to further test this model.

## Introduction

 The incidence of breast cancer (BC) is increasing among young women,^1^ and the average age of childbirth has increased; therefore, many BC survivors have not yet fulfilled their family planning and desire to have children at the time of the diagnosis. However, a decrease in ovarian function occurs secondary to systemic therapy, and accordingly, fertility preservation procedures are proposed to patients before the initiation of cancer treatments.^2^ If one could predict which patients would not be infertile after BC treatment, these would be spared fertility preservation procedures and their likely hazards, including the delay in treatment initiation, the possible risk of cancer growth triggered by administered exogenous sex steroid compounds, and the costs.

 Anti-Müllerian hormone (AMH), secreted by the granulosa cells of the ovarian follicles, has been shown to be a highly sensitive marker for ovarian reserve.^3-5^ The number of follicles in the ovaries as counted under ultrasound, and known as antral follicle count (AFC), is another accurate measurement for fertility potential.^6^ AFC and AMH have been recognized as the best markers^7^ and the only accurate indicators^8^ of ovarian reserve in women. In comparison with AFC, AMH has been demonstrated to be a more cost-effective measurement^9^ and an even more accurate method.^10^ A recent systematic review^11^ clarified that post-treatment AMH is a useful marker for the evaluation of ovarian function and that it is directly linked with pre-treatment AMH.

 Previous studies have attempted to define a cut-off point for pre-treatment AMH^7^ or introduce a nomogram^12^ to foresee ovarian reserve and fertility status after BC management; however, clinicians are far from reaching a consensus on this matter, and targeted studies are needed to approach this concern.^7,13^ Therefore, a study was conducted to find a specific level of pre-treatment AMH and a combination model consisting of the patient’s features and AMH to predict and determine which patients need to undergo fertility preservation interventions.

## Materials and Methods

###  Study Design and Setting

 The study protocol was approved by the Deputy Director of Research of Tehran University of Medical Sciences (TUMS) and received the ethical approval of the Ethics Committee of TUMS (Ethics Code: IR.TUMS.VCR.REC.1397.242). The study was performed according to the ethical principles of the Declaration of Helsinki, and all the participants signed a written informed consent form prior to entering the study.

 This is a multicenter prospective cohort study. The study population consisted of women attending two referral centers affiliated with TUMS, including Arash Women’s Hospital and the Cancer Institute, from December 2018 to January 2020 and diagnosed as having BC based on histological assessments. All eligible women who visited during this period were considered potential participants.

###  Eligibility Criteria

 The inclusion criteria consisted of a newly diagnosed non-metastatic invasive BC, need for chemotherapy (ChT) during treatment, premenopausal status, age 45 or less, and willingness to participate in the study. On the other hand, the exclusion criteria included a history of infertility, previous ChT, exogenous sex hormone therapy or contraceptive use in the recent two years, and a history of abnormal uterine bleeding, polycystic ovarian disease, endometriosis, surgery of the ovary, liver, or renal failure.

###  Variables and Outcomes

 The main dependent variables were the pre-treatment AMH level (AMH0), one month (AMH1), and two-year post-treatment AMH level (AMH2). Other variables included AFC before (AFC0) and two years after (AFC2) treatment, the patient’s medical history and demographic, anthropometric, and reproductive features, tumor characteristics, type of adjuvant treatment, and the status of the patient’s menstruation during the study.

 The AMH levels were very low in the measurements conducted one month after the end of ChT.^14^ Therefore, AMH1 was not considered in the analysis. The primary outcome consisted of changes in AMH levels before and two years after treatment (∆AMH) relative to AMH0 and other inherent features of the patients. The secondary outcomes included the amenorrhea status and AFC two years after treatment and their relationship with AMH0.

###  Measurements

 Data regarding age, reproductive factors, and past medical history were collected via an interview held with every participant by trained staff in each center at the entry to the study, and height and weight were measured and recorded. The data about menstruation and amenorrhea were gathered and recorded at entry and at each subsequent visit.

 The blood samples were drawn at three time points (at entry, one month, and 2 years after the end of ChT); sera were separated and carried in a cold pack to the laboratory. The AMH level was measured by enzyme-linked immunosorbent assay (Beckman Coulter, AMH gene II assay, Brea, CA, USA kit), with intra- and inter-assay variations of 7.7% and a 0.08 ng/mL limit of detection. An AMH2 above 0.7 ng/mL was considered acceptable.^15^

 All AFCs were conducted by an experienced infertility gynecologist in one center and by an experienced women radiologist in the other through performing transvaginal ultrasound with a C9-4V-MHz probe (Philips affinity 50) and counting the number of antral follicles 2‒10 mm in size in transverse and longitudinal planes; the follicular size was calculated as the average number of the two perpendicular dimensions of each follicle, including the largest diameter.

###  Bias

 To address the possibility of detection bias, AMH measurement kits were bought from one provider, and measurements were performed in one center. In addition, for the sake of adjusting the AFC results obtained in the two centers, the two specialists had matched their performance at the time of designing the study before the initialization of the project. The patients who had recurrences and underwent additional systemic therapy during the follow-up were withdrawn from the analysis to eliminate the probable bias of these treatments.

###  Sample Size

 To calculate the sample size, we considered the figures reported by Anderson et al,^16^ where the AMH before and two years after treatment were 1.29 ± 0.21 and 0.32 ± 0.07 ng/mL, respectively. Considering an α = 0.05 and β = 20%, at least 50 patients were needed to find the above difference between before and after treatment AMH levels using the Epi Info calculator (http://www.openepi.com/SampleSize/SSMean.htm).

###  Statistical Methods

 The analyses of the descriptive variables were performed by SPSS, version 24 (IBM Corp., 2016; IBM SPSS Statistics for Windows, version 24.0; Armonk, NY: IBM Corp.). The data are presented as means ± standard deviations, as well as numbers and percentages for continuous and categorical variables, respectively. Continuous variables were compared using a *t* test, paired *t* test, and Wilcoxon ranked test based on normality and dependency of variables. Further, categorical variables were compared using the chi-square test. The normality of the AMH levels and AFC were assessed using the Kolmogorov-Smirnov test. Pearson or Spearman Correlation test was used to find the correlation between continuous variables. The ∆AMH level was obtained from the difference between AMH baseline and AMH after 2 years (AM0-AMH2). The AFC total was obtained from the sum of the right and left ovarian follicles.

 The following steps were performed to find the variables and cut-off points that could affect AMH2:

1. AMH0, body mass index (BMI), and age, according to the study by Su et al,^17^ were considered the effective variables. A receiver-operating-characteristic (ROC) curve analysis was performed for each parameter. The area under the ROC curve (AUC), sensitivity, and specificity are reported.
2. Multivariate logistic regression was performed for the relationship between effective variables and AMH2. The adjusted odds ratios (ORs) and 95% confidence intervals (CIs) are reported.
3. Python language via the sklearn library was used, along with two machine learning algorithms, including a top-down decision tree (DT) and random forest. AMH2 was considered the response variable, and classes were defined as < 0.7 or ≥ 0.7 ng/mL.^15,17^ The dataset was imbalanced, as more than 80% of the samples were in the first class. Therefore, the method of Chawla et al^18^ was to produce synthetic samples based on the real dataset. Age and three sex-hormone-dependent variables that are present in both parous and not-parous women were selected as predictors in our models; these included age at menarche (above or below 14^19^), menstruation pattern (regular or irregular), and AMH0. The overall reduction in node impurities as calculated by the Gini index was considered to evaluate the relative importance of each variable, and 5-fold cross-validation was used for both DT and random forest, choosing 20% of the dataset as test and 80% as training data. The mean of each metric was reported for both models as a final score. Accuracy, precision, recall, F1-score, and ROC were employed as metrics to measure the quality of the models.

## Results

###  Flow of Participants

**Figure 1:**
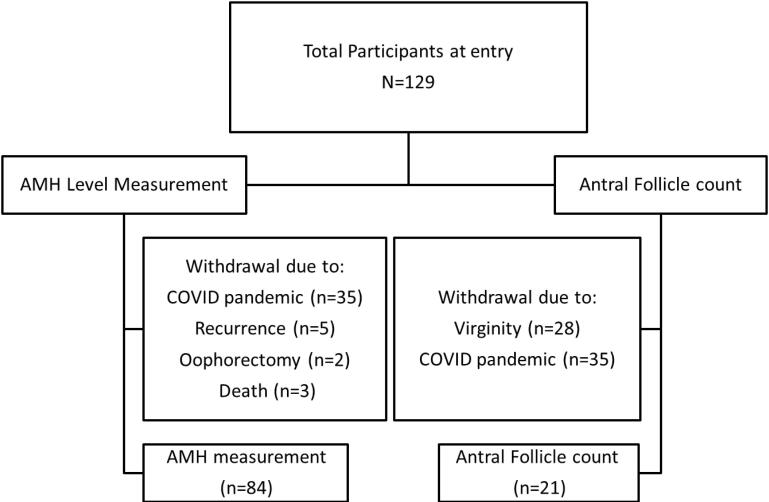
Flow Diagram of Participants

 Overall, 129 women were entered into the study. Of these, 45 patients withdrew or were withdrawn due to the COVID-19 pandemic (n = 35), recurrences and receiving new treatments (n = 5), oophorectomy (n = 2), and death (n = 3). Finally, the data of 84 patients were analyzed for this study. Sixty-three participants did not undergo AFC due to COVID-19 conditions (n = 35) and virginity (n = 28) and did not accept vaginal ultrasound. The flow of participants is depicted in Figure 1.

###  Descriptive Data

Table 1 provides the characteristics of all the participants and their tumors at entry and the types of systemic treatments according to the number of participants.

**Table 1:** Characteristics of All Participants and Their Tumors at Entry and Systemic Treatment Data (n = 84)

**Characteristics**	**Mean±SD or Number (Percent)**
Age	35.90 ± 5.15
BMI	25.14 ± 3.99
Age at menarche	13.43 ± 1.26
Gravidity	1.57 ± 1.41
Parity	1.16 ± 1.04
Abortion	0.43 ± 0.72
Tumor size (cm)	1.80 ± 0.73
Breastfeeding (months)	16.61 ± 18.62
Education	
University graduate	38 (45.2)
High school diploma	24 (28.6)
Below diploma	20 (23.8)
Missing	2 (2.4)
Job	
Housewife	55 (65.5)
Office worker	19 (22.6)
Medical staff	2 (2.4)
Others	8 (9.5)
Menstrual cycle	
Regular	71 (84.5)
Irregular	10 (11.9)
Missing	3 (3.6)
Family history of breast cancer	
Yes	13 (15.5)
No	71 (84.5)
Histologic type of tumor	
Invasive ductal carcinoma	78 (92.9)
Invasive lobular carcinoma	5 (6)
Missing	1 (1.2)
Lymph node involvement	
No	42 (50)
Yes	23 (7.4)
Unknown/Not applicable	19 (22.6)
Estrogen receptor	
Positive	62 (73.8)
Negative	15 (17.9)
Missing	7 (8.3)
Progesterone receptor	
Positive	58 (69)
Negative	19 (22.6)
Missing	7 (8.3)
HER2	
Negative	64 (76.2)
Positive	15 (17.9)
Missing	5 (6%)
Ki 67%	30.79 ± 19.91 (5–80%)
Grade	
1	15 (17.9)
2	34 (40.5)
3	15 (17.9)
Missing	20 (23.8)
Chemotherapy regimens	
ACT	66 (78.6)
Epirubicin CT	3 (3.6)
ACT-Carboplatin	2 (2.4)
5-FU-Epirubicin-Carboplatin	1 (1.2)
Paclitaxel-Carboplatin	6 (7.1)
Missing	6 (7.1)
Endocrine therapy regimens	
Tamoxifen	55(65.4)
Letrozole	5 (6)
Tamoxifen and letrozole	2 (2.4)
Exemestane	1 (1.2)
No	13 (15.5)
Missing	8 (9.5)
LHRH/GnRH analogue^*^	
Yes	18 (21.4)
No	54 (64.3)
Missing	12 (14.3)

*Note*. ^*^Among those receiving endocrine therapy. 5-FU: 5-fluorouracil; ACT: Doxorubicin, cyclophosphamide, and paclitaxel; CT: Cyclophosphamide and paclitaxel; SD: Standard deviation; BMI: Body mass index; LHRH: Luteinizing hormone-releasing hormone; GnRH: Gonadotropin-releasing hormone; HER2: Human epidermal growth factor receptor 2; CT: Chemotherapy.

###  Outcome Data and Main Results

 AMH0 and AFC levels had a normal distribution; however, AMH2 was not normally distributed. AMH0 and AMH2, as continuous and categorical variables, and data about AFC are presented in Table 2. The data of ∆AMH and the status of menstrual cycles two years after treatment according to the systemic treatments are listed in Table 3. Overall, 38 (45.24%) women were amenorrheic two years after treatment.

**Table 2:** Levels of AMH (n = 84) and Antral Follicular Count (n = 21) Before and Two Years After Treatment

	**AMH0 (n=84)**	**AMH2 (n=84)**	***P*** ** Value**
AMH level	3.38 ± 3.03	0.63 ± 1.41	< 0.001^*^
AMH category			< 0.001
Normal (≥ 0.7 ng/mL)	69 (82.1%)	14 (16.7%)	
Low (< 0.7 ng/mL)	15 (17.9%)	70 (83.3%)	
	**AFC0 (n=21)**	**AFC2 (n=21)**	
Right ovary	3.90 ± 3.55	1.52 ± 1.47	0.01
Left ovary	4.05 ± 2.94	1.14 ± 1.39	0.001
Total AFC	7.95 ± 6.08	2.71 ± 2.69	< 0.001

*Note*. AMH, Anti-mullerian hormone; AFC, Antral follicle count.

**Table 3:** AMH Changes and Number of Participants With or Without Amenorrhea Two Years After Treatment According to Systemic Treatments

**Systemic Treatment**	**AMH0**	**AMH2**	**∆AMH**	***P*** **Value**^a^
Chemotherapy				
Taxanes, cyclophosphamide and an anthracycline (n = 72)	2.43 ± 2.73	0.69 ± 1.51	3.12 ± 2.62	< 0.001
Paclitaxel + Carboplatin (n = 6)	3.62 ± 3.58	0.38 ± 0.52	4 ± 3.79	0.06
*P* value^b^	0.32	0.61	0.60	
Endocrine therapy				
Yes (n = 63)	2.33 ± 2.78	0.66 ± 1.51	3 ± 2.68	< 0.001
No (n = 13)	4.35 ± 4.31	0.34 ± 0.41	4.69 ± 4.50	0.003
*P* value^b^	0.04	0.45	0.21	
GnRH/LHRH analog therapy (among those receiving endocrine therapy)				
Yes (n = 18)	2.63 ± 3.03	0.69 ± 2.01	3.32 ± 2.72	0.002
No (n = 43)	2.26 ± 2.76	0.68 ± 1.30	2.94 ± 2.72	< 0.001
*P* value^b^	0.65	0.98	0.62	
**Systemic treatment**	**Amenorrhea (n=38)**		**Menstruating (n=46)**	***P*** **Value**^c^
Chemotherapy				
Containing cyclophosphamide and an anthracycline	35 (100%)		37 (86%)	0.03
Other	0 (0%)		6 (14%)	
Endocrine therapy				
Yes	31 (86.1%)		32 (80%)	0.55
No	5 (13.9%)		8 (20%)	

*Note*. LHRH: Luteinizing hormone-releasing hormone; GnRH: Gonadotropin-releasing hormone.AMH0 = AMH before treatment; AMH2 = AMH two years after treatment; ∆AMH = AMH0-AMH2. ^a^*P* value refers to the comparison between pre-treatment AMH and AMH after 2 years (paired *t*-test).^b^* P* value refers to the *t *test. ^c^*P* value refers to the chi-square test.

 Regarding our primary outcomes, ROC curve analysis revealed that AMH0 was the best indicator for predicting AMH2 ≥ 0.7 (cut-off = 3 ng/mL, AUC = 0.69, 95% CI: 0.54‒0.84) with a sensitivity of 79% and a specificity of 60%. However, BMI (cut-off = 25 kg/m^2^, AUC = 0.60, 95% CI: 0.46‒0.73,) and age (cut-off = 33.5 years old, AUC = 0.35, 95% CI: 0.19‒0.50) with a sensitivity and a specificity of 71% and 57%, respectively, for BMI, and a sensitivity and specificity of 57% and 30%, respectively, for age, were inferior for predicting AMH2.

 The result of logistic regression analysis showed that only AMH0, with OR equal to 3.36 (95% CI = 1.35‒22.17, *P* = 0.02), remained a significant predictor of AMH2. Furthermore, BMI ≥ 25 was directly associated with AMH2 (OR = 3.36, 95% CI = 0.90‒12.48) with borderline significance (*P* = 0.07). However, there was no association between age and AMH2 (OR = 0.92, 95% CI = 0.25‒3.46, *P* = 0.91).

**Figure 2:**
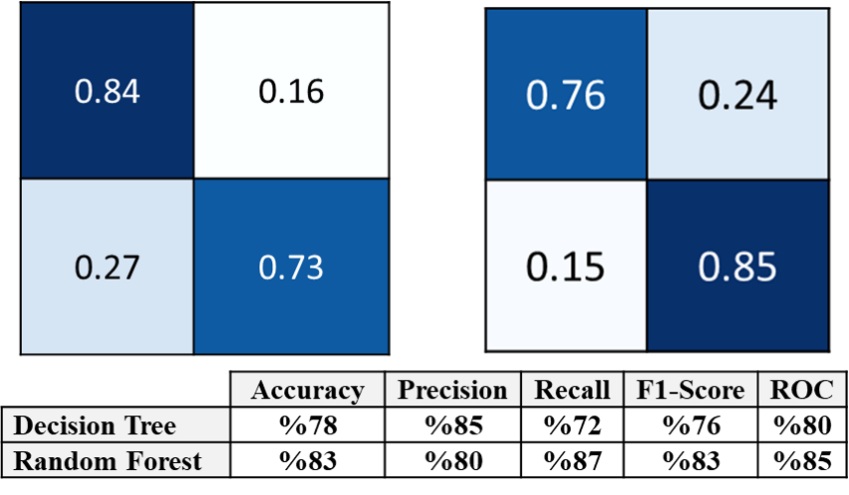
Confusion Matrixes of Decision Tree (The Above Row on the Right) and Random Forest (The Above on the Left), and the Models’ Validation Results (Below) for the Prediction of AMH Status Two Years After Breast Cancer Treatment, Considering AMH Before Chemotherapy, Age at Menarche, and Menstrual Patterns as Predictors in the Models. *Note*. The means of each metric for both models are reported as the final scores. Accuracy, precision, recall, F1-score, and ROC are used as metrics for measuring the quality of the models. The numbers are the means of 5-fold cross-validation’s results. ROC: Receiver operating characteristics; AMH: Anti-mullerian hormone

**Figure 3:**
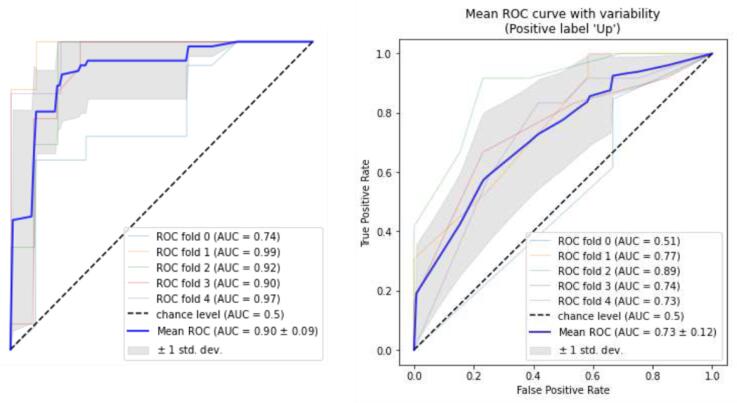
ROC Curves of Decision Tree (on the Right) and Random Forest (on the Left) for All 5-Fold Cross-Validation. *Note*. ROC: Receiver operating characteristics; AMH: Anti-mullerian hormone. The blue curves describe the means of 5-fold cross-validations. The area under the curve is 79% (on average) for the decision tree (Confidence interval = 85%) and 86% (on average) for the random forest (Confidence interval = 89%)

 The confusion matrices of the top-down DT and random forest and the results of the two models are displayed in Figure 2. The estimated means of the ROC curves for both models are illustrated in Figure 3. In summary, according to the DT, for an AMH0 above 3.3, an age less than 31, and a menarche age below 14, the patient will have a 93% probability of having an AMH2 above 0.7.

Considering the secondary outcomes, correlation analysis demonstrated that AFC0 was positively correlated with AMH0 (r = 0.127). In addition, AFC2 had a positive correlation with AMH0 (r = 0.095) and a negative correlation with ∆AMH (r = - 0. 047). Moreover, AFC0 (r = -0.041) and AFC2 (r = -0.036) had a weak negative correlation with age. None of these were significant due to the low number of AFC results. AMH levels before treatment were not statistically different considering amenorrhea status (2.94 ± 2.96 vs. 3.73 ± 3.07, *P* = 0.24).

## Discussion

 This study was conducted to determine pre-treatment AMH levels and produce a combination model that could predict an appropriate post-treatment ovarian reserve in premenopausal early BC patients. It was found that an AMH0 above 3 was the most reliable pre-ChT feature for the prediction of an AMH2 above 0.7 and that the best combination model was AMH > 3.3, age < 31, and menarche age < 14.

 The decreased rate of fertility after cancer treatment is mostly due to ChT,^2,20^ and various ChTs induce different levels of follicular depletion^21^; the worst are alkylating agents,^22^ followed by anthracyclines and taxanes, which are commonly administered for BC treatment. Most of our patients received ChT regimens containing anthracyclines and alkylating agents, so the sub-analysis of AMH2 did not yield significant results. However, amenorrhea was significantly more common in women receiving these compounds.

 Some studies^5,23,24^ defined the course of AMH level changes during and after ChT for BC. They reported a severe AMH decline during and immediately after ChT, with a subsequent rise after 2 to 4 years. Partridge et al^13^ compared post-ChT AMH and AFC of 20 BC patients with matched controls and found significantly lower levels due to ChT. In our study, the levels of AMH one month after ChT (AMH1) were extremely low, the AMH and AFC decreased significantly two years after BC treatment (*P* < 0.001 for both), and a high rate of amenorrhea (45.24%) was observed without any relation to the menstrual pattern at entry.

 Several studies^16,25,26^ have considered amenorrhea (vs. continuing menses) as the marker of ovarian dysfunction after BC treatment and investigated its relation to AMH0. They found that a lower AMH0 is a marker of amenorrhea after ChT. Anderson et al^27^ examined 75 BC patients and categorized AMH0 as low, medium, and high. They concluded that the former and latter groups were associated with amenorrhea and the return of menses two years after treatment, respectively. Su et al^17^ aimed to find a cut-off point for AMH0 that could predict the return of menses after treatment and found a level above 0.7 ng/mL in conjunction with a BMI>25 Kg/m^2^ and an age < 40 years. The results of these studies are quite in accordance with ours, and the results for the low AMH group of Anderson et al^27^ are mostly similar to our results. Likewise, the overall results of Su et al^17^ confirm ours, except that the cut-off point detected was extremely lower (0,7 vs. around 3 ng/mL), and we did not find BMI to be a sensitive and specific marker. However, the main limitation of their study is that the median time of patient follow-up was around 5 months, and the shortest interval was 3 months, which is a too short interval for the assessment of post-treatment amenorrhea. Additionally, as in all those studies, amenorrhea was considered a measure of ovarian function, while regular menses are not an appropriate marker of ovarian reserve.^5,28,29^ D’Avila et al^7^ followed a cohort of 52 BC patients and assessed AMH and AFC before and 6 months after receiving cyclophosphamide. They reported that cases ≥ 32 years of age with an AMH < 3.3 and an AFC < 13 had a higher probability of becoming amenorrheic; we will later discuss these results further.

 Similar to our study, other studies have considered post-treatment AMH as an accurate marker of ovarian function. To note, in our study, AMH2 = 0.7 was envisaged as the appropriate predictor of a good ovarian reserve; this value remains quite arbitrary but acceptable considering the present data.^15,17^ Henry et al^28^ studied 26 patients less than 50 years of age and found a positive and negative predictive value of around 95% and 86%, respectively, for AMH0 as a predictor of post-treatment ovarian reserve. However, they only envisaged detectable levels of AMH. Dillon et al^29^ concluded that AMH0 affected both the level and the speed of recovery of AMH without exploring a specific cut-off point. Anderson et al^30^ evaluated BC patients between 40 and 45 years of age and, while emphasizing the validity of post-treatment AMH as a marker for ovarian reserve, showed that AMH0 was a significant predictor of AMH after 30 months. This study mainly focused on the prediction of later ovarian function based on the AMH levels 6 months after treatment, which proved to be an accurate marker in this regard. The level of AMH0, which could be regarded as a safe predicting figure, was not explored in that study. Barnabei et al^12^ performed a meta-analysis and designed a nomogram, including age and AMH0, that could assist in the prediction of ovarian function after BC treatment. This nomogram yields results that are highly similar to ours, as an AMH0 around 3 would necessitate an age around 30 to produce an acceptable probability for a good ovarian reserve after treatment. Other possibilities are also detectable on the nomogram, but they mostly dictate a too high AMH0 or a very young age, which are extremely uncommon in BC. The results of D’Avila et al^7^ are in line with those of ours, although we could not assess AFC because of the low sample size. While our study had some advantages, including a larger sample size, consideration of AMH2 as a marker of ovarian function (vs. amenorrhea in their study), and a longer sensible follow-up, our studies yielded close results regarding the cut-off point for AMH0 (around 3) and age (31‒33 years) as the predictors of an acceptable ovarian reserve after treatment.

 During this study, the COVID-19 outbreak happened, which affected greatly our breast clinic schedules and the timing of the follow-up visits, as well as the method of visiting cancer patients (virtual vs. in-person visits), and therefore we could not ask patients to attend just for the sake of study tests. This led to the loss of patients for AFC2 (Figure 1). As a result, we could not obtain significant results for this variable, but we could show that AFC and AMH before any treatment were in accordance and that the recovery of AFC was better in women with higher AMH0 who had a lower decline in AMH2 (had a smaller ∆AMH).

 In our study, several methods were used to assess our main goal and define the cut-off point for AMH0. The results were reciprocally confirmative, emphasizing their consistency. However, this study had some limitations, including the inclusion of BC tumors with different molecular subtypes, the small number of women with AMH2 higher than 0.7, and the similarity of ChT regimens in most patients, which prohibited an efficient sub-analysis of the data.

## Conclusion

 The findings of this study revealed that fertility preservation before the initiation of treatments in premenopausal BC patients can be withheld in patients with an AMH0 above 3.3 if they were less than 31 years old and had their menarche before the age of 14. In addition, it was found that an AMH0 above 3 in a non-obese woman might be an acceptable threshold for the prediction of a functioning ovary two years after BC treatment.

## References

[1] Thomas A, Rhoads A, Pinkerton E, Schroeder MC, Conway KM, Hundley WG (2019). Incidence and survival among young women with stage I-III breast cancer: SEER 2000-2015. JNCI Cancer Spectr.

[2] Bjelic-Radisic V, Esfandbod M, Alipour S. Pregnancy in breast cancer survivors. In: Diseases of the Breast during Pregnancy and Lactation. 1st ed. Switzerland: Springer; 2020. p. 165-74. 10.1007/978-3-030-41596-9_23 32816278

[3] Dewailly D, Andersen CY, Balen A, Broekmans F, Dilaver N, Fanchin R (2014). The physiology and clinical utility of anti-Müllerian hormone in women. Hum Reprod Update.

[4] Broer SL, Broekmans FJ, Laven JS, Fauser BC (2014). Anti-Müllerian hormone: ovarian reserve testing and its potential clinical implications. Hum Reprod Update.

[5] Peigné M, Decanter C (2014). Serum AMH level as a marker of acute and long-term effects of chemotherapy on the ovarian follicular content: a systematic review. Reprod Biol Endocrinol.

[6] Broer SL, Mol BW, Hendriks D, Broekmans FJ (2009). The role of anti-Müllerian hormone in prediction of outcome after IVF: comparison with the antral follicle count. Fertil Steril.

[7] D’Avila ÂM, Biolchi V, Capp E, Corleta H (2015). Age, anti-Müllerian hormone, antral follicles count to predict amenorrhea or oligomenorrhea after chemotherapy with cyclophosphamide. J Ovarian Res.

[8] Ruddy KJ, O’Neill A, Miller KD, Schneider BP, Baker E, Sparano JA (2014). Biomarker prediction of chemotherapy-related amenorrhea in premenopausal women with breast cancer participating in E5103. Breast Cancer Res Treat.

[9] Rosen MP, Johnstone E, McCulloch CE, Schuh-Huerta SM, Sternfeld B, Reijo-Pera RA (2012). A characterization of the relationship of ovarian reserve markers with age. Fertil Steril.

[10] Knauff EA, Eijkemans MJ, Lambalk CB, ten Kate-Booij MJ, Hoek A, Beerendonk CC (2009). Anti-Müllerian hormone, inhibin B, and antral follicle count in young women with ovarian failure. J Clin Endocrinol Metab.

[11] Anderson RA, Cameron D, Clatot F, Demeestere I, Lambertini M, Nelson SM (2022). Anti-Müllerian hormone as a marker of ovarian reserve and premature ovarian insufficiency in children and women with cancer: a systematic review. Hum Reprod Update.

[12] Barnabei A, Strigari L, Marchetti P, Sini V, De Vecchis L, Corsello SM (2015). Predicting ovarian activity in women affected by early breast cancer: a meta-analysis-based nomogram. Oncologist.

[13] Partridge AH, Ruddy KJ, Gelber S, Schapira L, Abusief M, Meyer M (2010). Ovarian reserve in women who remain premenopausal after chemotherapy for early-stage breast cancer. Fertil Steril.

[14] Eslami B, Jalaeefar A, Moini A, Omranipour R, Haghighi M, Alipour S (2020). Significant post-chemotherapy decrease of ovarian reserve in Iranian women with breast cancer. Acta Med Iran.

[15] Lin C, Jing M, Zhu W, Tu X, Chen Q, Wang X (2021). The value of anti-Müllerian hormone in the prediction of spontaneous pregnancy: a systematic review and meta-analysis. Front Endocrinol (Lausanne).

[16] Anderson RA, Cameron DA (2011). Pretreatment serum anti-Müllerian hormone predicts long-term ovarian function and bone mass after chemotherapy for early breast cancer. J Clin Endocrinol Metab.

[17] Su HC, Haunschild C, Chung K, Komrokian S, Boles S, Sammel MD (2014). Prechemotherapy anti-Müllerian hormone, age, and body size predict timing of return of ovarian function in young breast cancer patients. Cancer.

[18] Chawla NV, Bowyer KW, Hall LO, Kegelmeyer WP (2002). SMOTE: synthetic minority over-sampling technique. J Artif Intell Res.

[19] Komura H, Miyake A, Chen CF, Tanizawa O, Yoshikawa H (1992). Relationship of age at menarche and subsequent fertility. Eur J Obstet Gynecol Reprod Biol.

[20] Dinas KD. Fertility counseling and preservation for breast cancer patients. In: Diseases of the Breast during Pregnancy and Lactation. 1st ed. Switzerland: Springer; 2020. p. 181-7. 10.1007/978-3-030-41596-9_25 32816280

[21] Blumenfeld Z (2012). Chemotherapy and fertility. Best Pract Res Clin Obstet Gynaecol.

[22] Lutchman Singh K, Davies M, Chatterjee R (2005). Fertility in female cancer survivors: pathophysiology, preservation and the role of ovarian reserve testing. Hum Reprod Update.

[23] Dezellus A, Barriere P, Campone M, Lemanski C, Vanlemmens L, Mignot L (2017). Prospective evaluation of serum anti-Müllerian hormone dynamics in 250 women of reproductive age treated with chemotherapy for breast cancer. Eur J Cancer.

[24] Zhou B, Kwan B, Desai MJ, Nalawade V, Ruddy KJ, Nathan PC (2022). Long-term anti-Müllerian hormone patterns differ by cancer treatment exposures in young breast cancer survivors. Fertil Steril.

[25] Anders C, Marcom PK, Peterson B, Gu L, Unruhe S, Welch R (2008). A pilot study of predictive markers of chemotherapy-related amenorrhea among premenopausal women with early-stage breast cancer. Cancer Invest.

[26] Su HI, Sammel MD, Green J, Velders L, Stankiewicz C, Matro J (2010). Anti-Müllerian hormone and inhibin B are hormone measures of ovarian function in late reproductive-aged breast cancer survivors. Cancer.

[27] Anderson RA, Rosendahl M, Kelsey TW, Cameron DA (2013). Pretreatment anti-Müllerian hormone predicts for loss of ovarian function after chemotherapy for early breast cancer. Eur J Cancer.

[28] Henry NL, Xia R, Schott AF, McConnell D, Banerjee M, Hayes DF (2014). Prediction of postchemotherapy ovarian function using markers of ovarian reserve. Oncologist.

[29] Dillon KE, Sammel MD, Prewitt M, Ginsberg JP, Walker D, Mersereau JE (2013). Pretreatment anti-Müllerian hormone levels determine rate of posttherapy ovarian reserve recovery: acute changes in ovarian reserve during and after chemotherapy. Fertil Steril.

[30] Anderson RA, Kelsey TW, Perdrix A, Olympios N, Duhamel O, Lambertini M (2022). Diagnostic and predictive accuracy of anti-Müllerian hormone for ovarian function after chemotherapy in premenopausal women with early breast cancer. Breast Cancer Res Treat.

